# The Treatment of Symptoms

**Published:** 1899-06-03

**Authors:** 


### The Treatment of Symptoms.

To the man in the street it would never occur that there could be any doubt as to the propriety of treating symptoms. Why else indeed, he would ask, does a patient go to a doctor if not to get relief from the pain he feels or the symptom from which he suffers? To the medical man fresh from a modern school, however, the matter appears in a very different light. All the teaching which he has received has led him to look upon the disease, and not the symptom, as the thing which has to be considered. Nay, it has generally gone even further, and has taught him, in treating disease, not to attend too exclusively to the nature of the malady, but above all things to have regard to the patient himself, his constitution, and his surroundings. Thus the poor "symptom" gets pushed far into the background, and the patient, seeking relief from his present trouble, does not always get exactly what he wants. He may get thoroughly "looked through;" his lungs, his heart, his liver may be investigated; even his ancestry may be inquired into; and a line of medicinal and dietetic regimen may be laid down for him, by following which he will have the best chance of being restored to such a degree of health as may be attainable. But when, after all this, the patient turns upon the doctor and says, "But what about this pain?" it is too apt to become clear that what has been in the doctor's mind has not been the pain at all, but its cause and origin, and the condition of system and of life that has made the occurrence of such pain possible.

This, we take it, is at least one of the causes which co-operate to make many well-to-do and even well-educated persons fly to chemists and such like people in their minor ailments rather than appeal at once to medical men. Such is the fulness and hurry of life, and it may be added such is man's confidence in his own constitution and power of recovery, that when he finds himself, as he would describe it, "out of sorts," he does not want to be thoroughly examined, and nothing is further from his desires than to have his family tendencies inquired into. All he asks for is present relief—a bottle that will take away the pain. Unfortunately, the more conscientious, the more fully educated, and the more desirous to do his best the doctor is, the more does he squirm at being asked to treat disease in such a fashion—"like a mere chemist." The result is very unfortunate. The country is flooded with physicians, careful and intelligent investigators of abstruse and difficult questions in medicine, men who are anxious above all things to get to the bottom of every case that presents itself before them, but not always men who are willing to bring their minds to bear upon the problem of making a smooth and easily taken bismuth mixture, or of deciding the delicate question whether syrup or glycerine or treacle forms the best sweetener for a cough medicine. The contempt with which the treatment of "mere symptoms" is held in the schools, and therefore in those who come from the schools, and the disdain with which medical men are coming to regard all that has to do with the making of physic and the pounding of drugs (which is perhaps but a natural consequence of their having been taught exclusively by men of consulting rank, and of having throughout their student life looked upon consulting work as their ideal, and, indeed, as the only thing worth working for), are, it is to be feared, having an influence which is likely in the end to be most injurious both to the public and the profession. The treatment of tuberculosis may be taken as a case in point. If a pallid-looking person applies to a conscientious medical man for something to relieve his cough the chances are that he gets thoroughly examined, that he is plied with most careful directions as to the treatment of his expectoration, and that he gets much good advice as to his mode of life, and perhaps as to the choice of a sanatorium. Incidentally, he gets a cough mixture. Now all this is very right and proper, but as a fact he does not care much about these things. He probably does not believe in his inmost heart that he really is consumptive—if he did he would go to a specialist—what he asked for was relief for his cough, and he is pretty sure to measure up the doctor by his power of giving him what he asked for. This is what we have to face. Our patients do not care a brass farthing whether they go to see doctors, chemists, quacks, or old women, so only that they get what they want; and what they want in nine cases out of ten is relief from the thing of which they complain. They measure us up not by our knowledge but by our success—by our success in small things—and that being so, however scientific we may be, and however much we may strive after the higher walks of medicine, the modern contempt for the treatment of symptoms and the modern disdain for the minutiæ of physic making which is continually cropping up in clinical teaching is childish if not worse. From a purely scientific and far-off point of view no doubt sick folk are provided by a kind Providence for better and more useful purposes than that of merely being relieved of their troubles. But then that is not the patient's point of view.

